# Sexual dimorphism in aging hematopoiesis: an earlier decline of hematopoietic stem and progenitor cells in male than female mice

**DOI:** 10.18632/aging.202167

**Published:** 2020-12-09

**Authors:** Eui-Young So, Euy-Myoung Jeong, Keith Q. Wu, Patrycja M. Dubielecka, Anthony M. Reginato, Peter J. Quesenberry, Olin D. Liang

**Affiliations:** 1 Division of Hematology/Oncology, Department of Medicine, Rhode Island Hospital, Warren Alpert Medical School of Brown University, Providence, Rhode Island 02903, USA; 2 Division of Rheumatology, Department of Medicine, Rhode Island Hospital, Warren Alpert Medical School of Brown University, Providence, Rhode Island 02903, USA

**Keywords:** aging, hematopoietic stem and progenitor cell, sexual dimorphism, sex hormone

## Abstract

Adult hematopoietic stem and progenitor cells (HSPCs) reside in the bone marrow (BM) ensuring homeostasis of blood production and immune response throughout life. Sex differences in immunocompetence and mortality are well-documented in humans. However, whether HSPCs behave dimorphically between sexes during aging remains unknown. Here, we show that a significant expansion of BM-derived HSPCs occurs in the middle age of female but in the old age of male mice. We then show that a decline of HSPCs in male mice, as indicated by the expression levels of select hematopoietic genes, occurs much earlier in the aging process than that in female mice. Sex-mismatched heterochronic BM transplantations indicate that the middle-aged female BM microenvironment plays a pivotal role in sustaining hematopoietic gene expression during aging. Furthermore, a higher concentration of the pituitary sex hormone follicle-stimulating hormone (FSH) in the serum and a concomitant higher expression of its receptor on HSPCs in the middle-aged and old female mice than age-matched male mice, suggests that FSH may contribute to the sexual dimorphism in aging hematopoiesis. Our study reveals that HSPCs in the BM niches are possibly regulated in a sex-specific manner and influenced differently by sex hormones during aging hematopoiesis.

## INTRODUCTION

Aging in humans affects systemic homeostasis, reduces the responsiveness to physical and mental challenges, increases the vulnerability to disease and death. Immunological mechanisms are known to be involved in the aging process. Accumulating evidence suggests that efforts to control inflammatory status may allow a better chance of successful aging whereas pro-inflammatory genotypes are related to unsuccessful aging [1–5]. Sex differences in immunocompetence and mortality are well-documented in humans [6]. Women in most societies not only have longer lifespans, they also are more resilient against infectious and some non-infectious diseases such as cancer [7], but women are often more susceptible to autoimmune diseases [8, 9]. Possible causes for these sex disparities include X chromosome-linked genetic factors [10–12], regulation of the immune response through sex hormones, and sex-dependent differences in telomere attrition [11, 13]. The X chromosome contains a large number of immune-related genes. As such, differential expression of X chromosome-linked immune regulatory genes between males and females could potentially contribute to sex disparities in immune responses important for the susceptibility of infectious disease, development of autoimmunity and cancer [14]. Gonadal sex hormones (SexHs) are divided into three classes: androgens (mainly testosterone), estrogens (mainly estradiol), and progesterone. In men, plasma testosterone concentrations remain stable almost up to 60 years of age and decline with aging. But in women there is a fluctuation in estrogen and progesterone due to variation in concentrations of the pituitary SexHs luteinizing hormone (LH) and follicle-stimulating hormone (FSH) during menstrual cycle [15]. The SexHs exert their biological functions through hormone receptors expressed by different types of immune cells. Estrogens promote while androgens suppress immune responses during infections, after vaccination or in the case of autoimmunity [16]. The aging process is also known to affect the sexual dimorphism in immune response and disease susceptibility. Although males and females experience the same types of aging-related changes to the immune system, males experience them earlier or more dramatically than females [17–19]. Both the development of immune cells from hematopoietic stem and progenitor cells (HSPCs) and the function of immune cells following terminal differentiation are affected by aging. Thus far, there are very few studies directly comparing age-related changes of the differentiated immune cells between males and females [18, 20, 21], and despite the seemingly interrelationship between sex, aging and immunity, little is known whether aging affects the immune system differently between males and females at the hematopoietic stem and progenitor cell level.

HSPCs reside in the bone marrow (BM) and ensure homeostatic production of hematopoietic cells and immune response throughout life. Though many hematopoietic cell types including HSPCs have been shown to respond to sex hormones [16, 22–25], it is still very common that studies on HSPCs have been performed on only one sex or analyzed without distinguishing between sexes. Recently, Nakada et al. showed that hematopoietic stem cells (HSCs) respond to estrogen and divide more frequently in female than in male mice, resulting in enhanced self-renewal and augmented erythropoiesis [26]. Interestingly, the increased cell cycle activity of HSCs exposed to higher level of estrogen would also involve potentially higher risk for mutations during cell divisions, susceptibility to genotoxic agents, and accelerated aging. Indeed, estradiol treated HSPCs appeared to become exhausted sooner in serial transplantation experiments [27]. However, whether HSPCs function similarly or differently between the sexes during aging is not known. Thus, the objective of the current study was to determine whether such a sexual dimorphism exists in aging hematopoiesis and whether sex hormones play a role. To investigate HSPC function, we performed a series of analysis on hematopoietic gene expression in the lineage-Sca-1+c-kit+ (LSK) cells isolated from BM of both male and female mice of different age groups: young adults (11-13 weeks old), middle-aged (60-70 weeks old) and old (85-90 weeks old) [28]. The BM-derived LSK cells were known to have enriched numbers of HSPCs in mice [29]. To assess the differences between male and female hematopoietic BM microenvironment in aging hematopoiesis, we also performed sex-mismatched BM transplantation. Furthermore, to investigate possible involvement of sex hormones on hematopoiesis during aging, we examined the gene expression levels for pituitary sex hormone follicle-stimulating hormone receptor (FSHR), luteinizing hormone receptor (LHR), and prolactin receptor (PRLR), as well as the gene expression levels for gonadal sex hormone estrogen receptors (ERα and ERβ), androgen receptor (AR) and progesterone receptor (PR) in the LSK cells isolated from BM of male and female mice of different age groups. We show that a decline of HSPCs occurs in male mice much earlier in the aging process than that in female mice. The concomitant higher hormone concentration and higher receptor expression of FSH, but not of any other sex hormones, in the middle-aged and old female mice than age-matched male mice suggests that FSH may contribute to the sexual dimorphism in aging hematopoiesis. Our study reveals that HSPCs in the BM niches are possibly regulated in a sex-specific manner and influenced differently by sex hormones during aging hematopoiesis.

## RESULTS

### A significant expansion of BM-derived HSPCs in the middle age of female but in the old age of male mice

**Figure 1:**
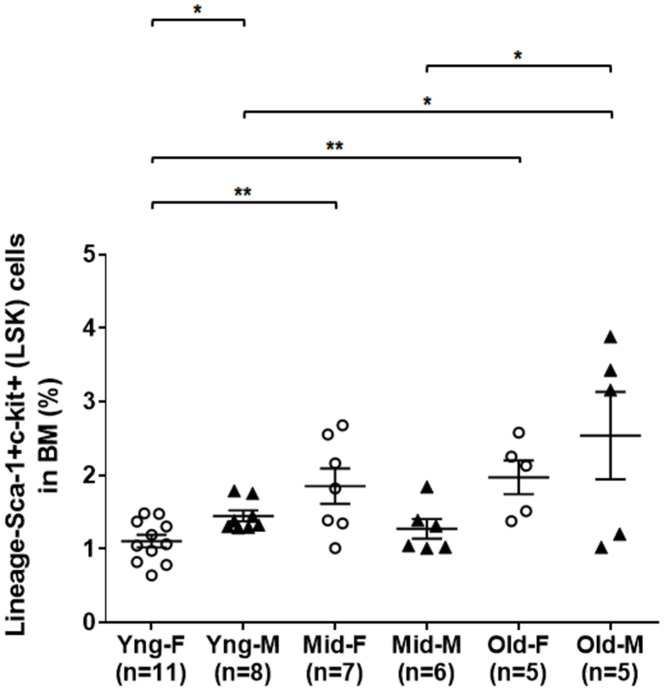
**A significant expansion of BM-derived HSPCs in the middle age of female but in the old age of male mice.** BM-derived lineage-Sca-1+c-kit+ (LSK) hematopoietic stem and progenitor cells were isolated via flow cytometry cell sorting, and percentages of the LSK cells among total live BM cells were compared between male and female of young, middle-aged and old mice. Multiple comparisons within 3 female groups and within 3 male groups were done separately using ordinary one-way ANOVA analysis; comparison between Yng-F and Yng-M was done using unpaired two-tailed Student’s t-test analysis. * P < 0.05, ** P < 0.01.

As shown in Figure 1, flow cytometry analysis indicated that the percentages of LSK cells in the BM of middle-aged female (Mid-F) and old female (Old-F) mice were significantly higher than that of young female (Yng-F) mice. The percentages of LSK cells were comparable between Mid-F and Old-F mice. However, the percentages of LSK cells were comparable between young male (Yng-M) and middle-aged male (Mid-M) mice. The percentage of LSK cells in old male mice (Old-M) was significantly higher than that of Yng-M or Mid-M mice. Interestingly, Yng-M mice appeared to have higher percentage of LSK cells in the BM than Yng-F. Thus, the number of HSPCs increases significantly from young age to middle age in female mice, whereas the number of HSPCs increases significantly from middle age to old age in male mice.

### An age-related down-regulation of hematopoietic gene expression in HSPCs of middle-aged male but not female mice

**Figure 2:**
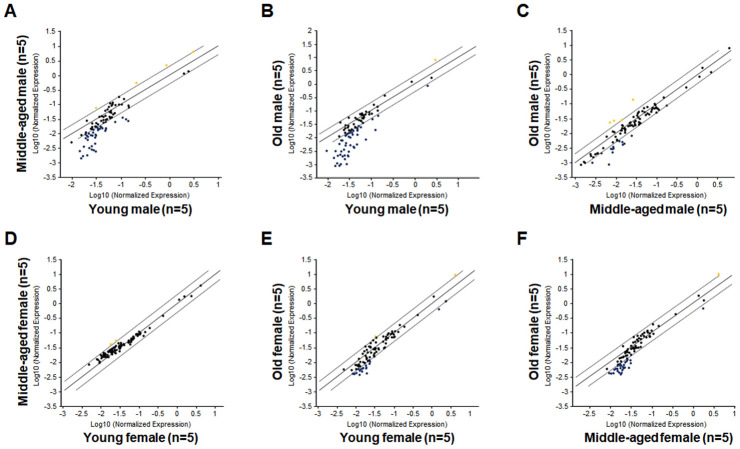
**Comparisons of relative hematopoietic gene expression in BM-derived LSK cells among young, middle-aged and old mice of both male and female mice.** (**A**) Comparison between middle-aged male and young male mice. (**B**) Comparison between old male and young male mice. (**C**) Comparison between old male and middle-aged male mice. (**D**) Comparison between middle-aged female and young female mice. (**E**) Comparison between old female and young female mice. (**F**) Comparison between old female and middle-aged female mice. Yellow dots indicate genes with potentially higher level of expression in mice of y-axis than that in mice of x-axis; black dots indicate genes with comparable level of expression; blue dots indicate genes with potentially lower level of expression in mice of y-axis than that in mice of x-axis.

As shown in Figure 2A and Table 1, a large number of hematopoietic genes have significantly down-regulated in the LSK cells of Mid-M mice compared with Yng-M mice. Similarly, a number of hematopoietic genes have significantly down-regulated in the LSK cells of Old-M mice compared with Yng-M mice (Figure 2B and Table 2). Several hematopoietic genes in the LSK cells showed consistently significant decline of relative gene expression from young to middle-aged and to old male mice (Figure 2C and Table 3). Strikingly, comparisons of the hematopoietic gene expression profiles among Yng-F, Mid-F and Old-F mice revealed a different pattern. There were no differences when the gene expression levels in the LSK cells of Mid-F mice compared with those of Yng-F mice (Figure 2D), which is a stark contrast to the comparison between Mid-M mice and Yng-M mice shown in Figure 2A. Further, there were a few genes showing a non-significant trend toward reduced expression when the gene expression profile in the LSK cells of Old-F mice compared with that of Yng-F mice (Figure 2E), which is again in stark contrast to the comparison between Old-M mice and Yng-M mice shown in Figure 2B. In addition, there were a few genes showing a non-significant trend toward reduced expression when the gene expression levels in the LSK cells of Old-F mice compared with those of Mid-F mice (Figure 2F), which is similar to the comparison between Old-M mice and Mid-M mice shown in Figure 2C. Combined, we found that an age-related and significant down-regulation of hematopoietic gene expression in HSPCs occurs in middle-aged male but not in middle-aged female mice.

**Table 1:** Preponderant decreases of relative gene expression in LSK cells of middle-aged male compared with young male mice.

**Gene Symbol**	**Fold Regulation (> 2-fold)**	**P Value (< 0.05)**
*Lmo2*	2.58	0.000353
*Angpt1*	2.38	0.000148
*Flt3l*	-2.01	0.045607
*Ccr1*	-2.07	0.046098
*Tlr3*	-2.27	0.049915
*Fut10*	-2.36	0.028103
*Lef1*	-2.6	0.014575
*Cd86*	-2.64	0.007944
*Il20*	-2.8	0.048758
*Kitl*	-2.81	0.024654
*Il31ra*	-3.12	0.032087
*Inhba*	-3.13	0.032466
*Jag1*	-3.15	0.029
*Lrmp*	-3.17	0.016812
*Hdac4*	-3.32	0.023868
*Ahsp*	-3.38	0.033585
Gata1*	-3.51	0.038088
*Pax5*	-3.58	0.015632
Fzd1*	-3.73	0.02228
*Inha*	-3.96	0.026484
*Tnfsf11*	-4.23	0.022865
Trim10*	-5.03	0.015697
Il10*	-5.82	0.012392
Il1a*	-6.75	0.013767
Il12b*	-6.99	0.011579
Notch4*	-7.2	0.011783
Il2*	-9.17	0.015044
Dll1*	-10.17	0.008511
Mal*	-10.65	0.010489
Il25*	-11.1	0.007668
Wnt3a*	-11.67	0.007274
Il11*	-12.82	0.009566

*Also appears in Table 4.

**Table 2:** Significant decreases of relative gene expression in LSK cells of old male compared with young male mice.

**Gene Symbol**	**Fold Regulation (> 2-fold)**	**P Value (< 0.05)**
*Map4k1*	-2.2	0.004889
*Mmp9*	-2.5	0.040668
*Cd14*	-2.95	0.029912
*Tlr3*	-3.08	0.033855
*Csf1*	-3.16	0.020331
*Socs5*	-3.45	0.032356
*Cd86*	-3.52	0.008358
*Tek*	-5.2	0.032358
*Cd4*	-7.14	0.035496
*Trim10*	-8.73	0.026001
*Cd80*	-9.1	0.028837
*Fzd1*	-10.94	0.047409
*Il25*	-18.58	0.045537
*Il20*	-22.39	0.034091

**Table 3:** Hematopoietic genes in LSK cells from male mice with consistently significant decline of relative gene expression throughout lifespan.

**Gene Symbol**	**Middle-aged vs Young**	**Old vs Young**
*Tlr3*	-2.27	-3.08
*Cd86*	-2.64	-3.52
*Trim10*	-5.03	-8.73
*Fzd1*	-3.73	-10.94
*Il25*	-11.1	-18.58

### A delayed decline in hematopoietic gene expression in female HSPCs during aging

**Figure 3:**
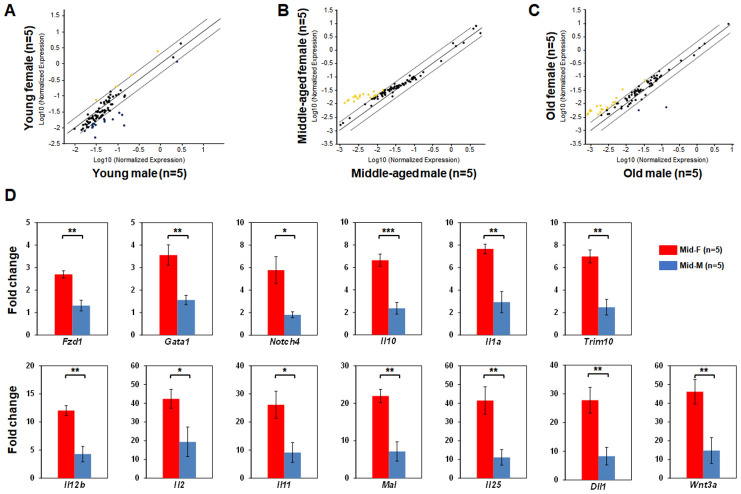
**Comparisons of relative hematopoietic gene expression in BM-derived LSK cells between male and female of young, middle-aged and old mice.** (**A**) Comparison between young female and young male mice. (**B**) Comparison between middle-aged female and middle-aged male mice. (**C**) Comparison between old female and old male mice. Yellow dots indicate genes with potentially higher level of expression in mice of y-axis than that in mice of x-axis; black dots indicate genes with comparable level of expression; blue dots indicate genes with potentially lower level of expression in mice of y-axis than that in mice of x-axis. (**D**) Real time PCR on individual genes represented by yellow dots from (**B**) was carried out to confirm that they had a significantly higher expression in LSK cells from middle-aged female than middle-aged male mice. * P < 0.05, ** P < 0.01, *** P < 0.001.

Next, we compared the LSK hematopoietic gene expression profiles between age-matched male and female mice. The LSK cells of Yng-F and Yng-M mice showed a comparable expression profile except for a few transcripts with a non-significant trend toward reduced expression in Yng-F (Figure 3A). However, a considerable number of hematopoietic genes in the LSK cells of Mid-F mice exhibited significantly higher expression than those in Mid-M mice (Figure 3B, Table 4). It is worth noting that all genes identified in Table 4, which had a higher expression level in Mid-F than Mid-M mice, were also identified in Table 1 as down-regulated genes in Mid-M compared with Yng-M mice. The LSK cells of Old-F and Old-M mice showed a mostly comparable expression profile except for several genes with a non-significant trend toward higher expression in Old-F than Old-M mice (Figure 3C). Using real time PCR, we could confirm in individual genes the higher gene expression levels in Mid-F than Mid-M mice (Figure 3D). Thus, our results indicate a delayed decline in hematopoietic gene expression in female compared with male HSPCs during aging.

**Table 4:** Significantly higher levels of relative gene expression in LSK cells of middle-aged female than middle-aged male mice.

**Gene Symbol**	**Fold Regulation (> 2-fold)**	**P Value (< 0.05)**
Fzd1*	3.37	0.000897
Gata1*	3.58	0.011052
Notch4*	4.82	0.015165
Il10*	4.85	0.000813
Il1a*	5.06	0.000726
Trim10*	5.13	0.000249
Il12b*	5.76	0.00124
Il2*	6.31	0.010433
Il11*	7.25	0.015972
Mal*	7.47	0.001869
Il25*	7.53	0.005712
Dll1*	7.63	0.010145
Wnt3a*	7.75	0.002092

*Also appears in Table 1.

### Middle-aged female BM microenvironment sustains hematopoietic gene expression during aging

**Figure 4:**
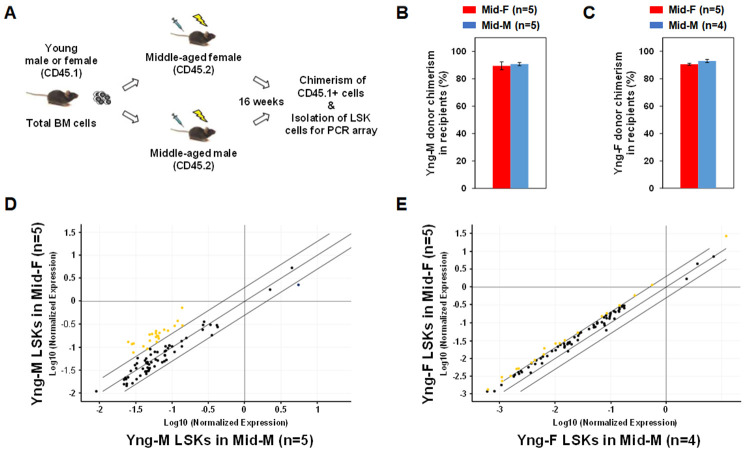
**Sex-mismatched heterochronic BM transplantations.** (**A**) Schematic of the experimental design of sex-mismatched heterochronic BM transplantations. Total BM cells were isolated from male and female CD45.1 young donor mice, and transplanted into lethally irradiated middle-aged female or male C57BL/6J recipient mice. After 16 weeks, CD45.1+ donor cell chimerism was determined and donor-derived lineage-Sca-1+c-kit+ (LSK) cells were isolated per flow cytometry cell sorting. (**B**) Yng-M donor cell chimerisms in Mid-F and Mid-M were comparable and ≥ 90%. (**C**) Yng-F donor cell chimerisms in Mid-F and Mid-M were also comparable and ≥ 90%. (**D**) A considerable number of hematopoietic genes (represented by yellow dots) in the Yng-M-derived LSK cells from the Mid-F recipients exhibited significantly higher expression than from the Mid-M recipients. (**E**) A number of hematopoietic genes (represented by yellow dots) in the Yng-F-derived LSK cells from the Mid-F recipients exhibited significantly higher expression than from the Mid-M recipients. Yellow dots indicate genes with potentially higher level of expression in mice of y-axis than that in mice of x-axis; black dots indicate genes with comparable level of expression; blue dots indicate genes with potentially lower level of expression in mice of y-axis than that in mice of x-axis.

In order to determine whether the middle-aged female BM microenvironment plays a role in sustaining hematopoietic gene expression during aging, we performed sex-mismatched heterochronic BM transplantations in mice. C57BL/6 mice with CD45.2 allele were used as recipients and injected with whole BM cells from congenic mice with CD45.1 allele so that donor or recipient cells can be readily identified by their CD45 allotypes. As illustrated in Figure 4A, whole BM cells from CD45.1 Yng-M or Yng-F mice were transplanted into lethally irradiated CD45.2 Mid-F or Mid-M mice. After 16 weeks, Yng-M CD45.1+ donor cell chimerism in both Mid-F and Mid-M recipients were ≥ 90% (Figure 4B). Similar results were obtained with Yng-F CD45.1+ donor cell chimerism in both Mid-F and Mid-M recipients (Figure 4C). Given the comparable chimerisms in the recipient mice, we then isolated the donor-derived LSK cells from the transplant recipients and performed a hematopoietic gene expression profiling on these cells. As shown in Figure 4D and Table 5, a considerable number of hematopoietic genes in the Yng-M-derived LSK cells isolated from the Mid-F recipients exhibited significantly higher expression than from the Mid-M recipients. Similarly, a number of hematopoietic genes in the Yng-F-derived LSK cells isolated from the Mid-F recipients exhibited significantly higher expression than from the Mid-M recipients (Figure 4E, Table 6). Combined, these results demonstrate that the middle-aged female BM microenvironment plays a pivotal role in sustaining hematopoietic gene expression during aging.

**Table 5:** Significantly higher levels of relative gene expression in the young male-derived LSK cells isolated from middle-aged female than from middle-aged male recipients.

**Gene Symbol**	**Fold Regulation (> 2-fold)**	**P Value (< 0.05)**
*Lef1*	2.05	0.041182
*Etv6*	2.15	0.045112
*Hdac7*	2.38	0.038185
*Map4k1*	2.62	0.029468
*Vav1*	2.63	0.028521
*Kit*	2.71	0.015660
*Cebpg*	2.72	0.046110
*Nos2*	2.81	0.026050
*Stat3*	2.84	0.042605
*Cd44*	3.11	0.027615
*Cd164*	3.40	0.018434
*Stat1*	3.69	0.020073
*Runx1*	3.89	0.019716
*Ets1*	4.12	0.005986
*Lmo2*	5.28	0.034616
*Ptprc*	5.30	0.007924

**Table 6:** Significantly higher levels of relative gene expression in the young female-derived LSK cells isolated from middle-aged female than from middle-aged male recipients.

**Gene Symbol**	**Fold Regulation (> 2-fold)**	**P Value (< 0.05)**
*Notch4*	2.05	0.015510
*Lmo2*	2.06	0.032751
*Inha*	2.10	0.032286
*Gata2*	2.17	0.017064
*Pf4*	2.18	0.048480
*Il25*	2.20	0.015219
*Cd3g*	2.21	0.029934
*B2m*	2.24	0.032040
*Tek*	2.38	0.022424
*Fzd1*	2.53	0.000638
*Cd3d*	2.59	0.016168
*Il10*	2.74	0.028577
*Tlr3*	3.02	0.020078

### Differential sex hormone receptor (SexHR) gene expression in BM-derived HSPCs of male and female mice during aging

**Figure 5:**
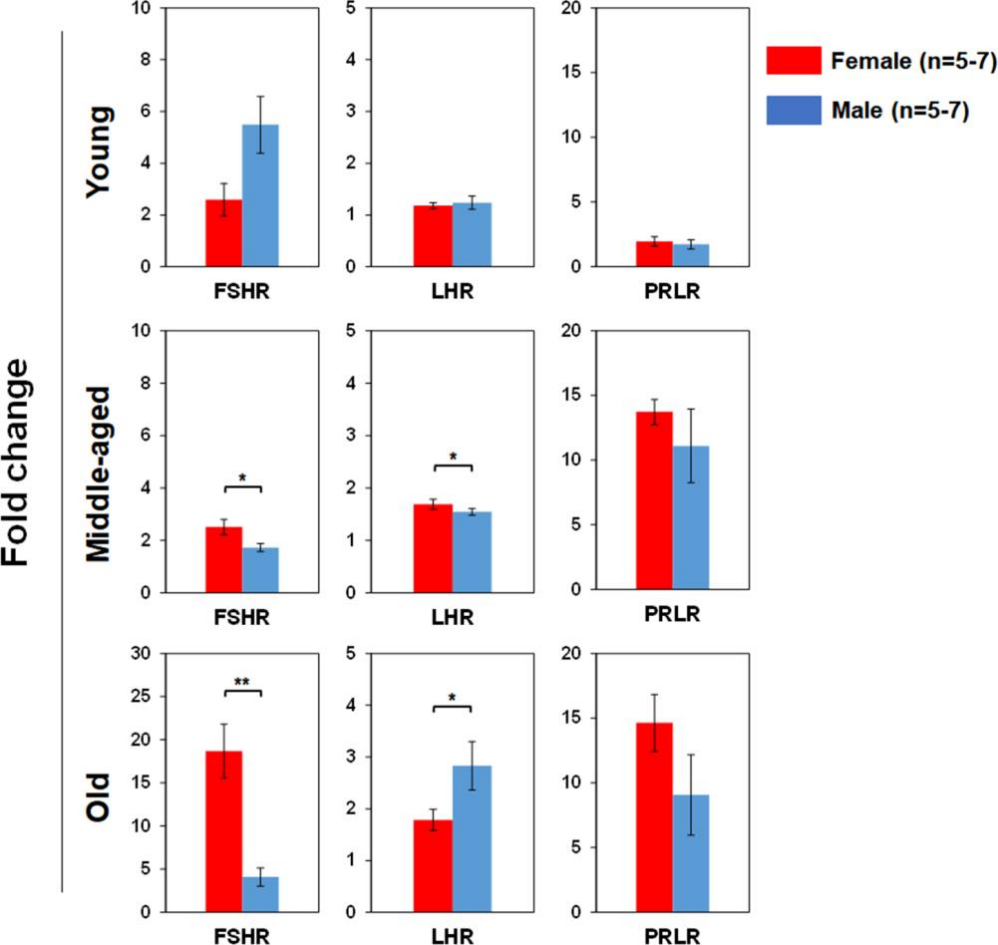
**Comparisons of relative pituitary SexHR gene expression between male and female of young, middle-aged and old mice.** Total RNA was purified from LSKs of indicated mice and subjected to real-time PCR to detect gene expression. *GAPDH* was used as a reference gene to normalize each gene expression. Left panels: FSHR gene expression between male and female of young, middle-aged and old mice. Middle panels: LHR gene expression between male and female of young, middle-aged and old mice. Right panels: PRLR gene expression between male and female of young, middle-aged and old mice. FSHR: follicle-stimulating hormone receptor, LHR: luteinizing hormone receptor, PRLR: prolactin receptor. * P < 0.05, ** P < 0.01.

**Figure 6:**
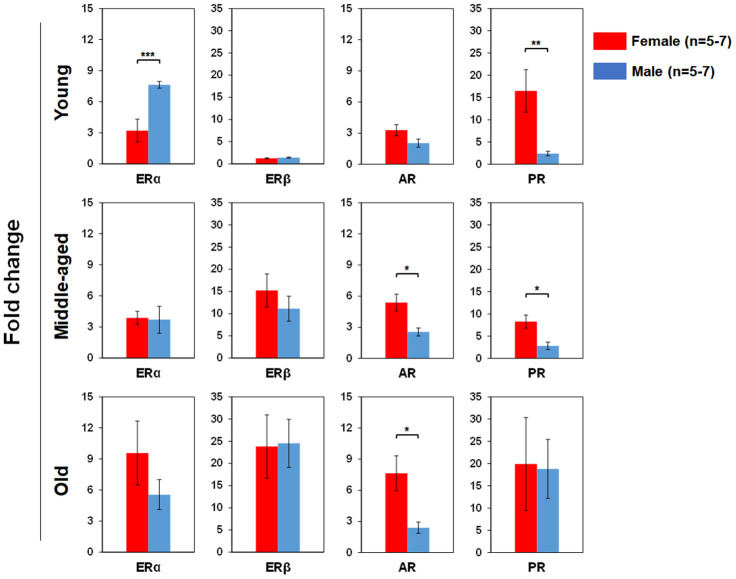
**Comparisons of relative gonadal SexHR gene expression between male and female of young, middle-aged and old mice.** Left panels: ERα gene expression between male and female of young, middle-aged and old mice. Middle-left panels: ERβ gene expression between male and female of young, middle-aged and old mice. Middle-right panels: AR gene expression between male and female of young, middle-aged and old mice. Right panels: PR gene expression between male and female of young, middle-aged and old mice. ERα: estrogen receptor α, ERβ: estrogen receptor β, AR: androgen receptor, and PR: progesterone receptor. * P < 0.05, ** P < 0.01, *** P < 0.001.

We used real time RT-PCR to determine pituitary SexHR gene expression in BM-derived LSK cells isolated from both male and female mice of different age groups (Figure 5). We found significantly higher FSHR gene expression in the LSK cells from middle-aged and old female than age-matched male mice. LHR gene expression in the LSK cells was higher in middle-age female than male, but higher in old male than female mice. PRLR gene expression in the LSK cells had no differences between male and female mice in all age groups. We also determined gonadal SexHR gene expression in BM-derived LSK cells isolated from both male and female mice of different age groups (Figure 6). We found significantly higher ERα expression in LSK cells of Yng-M mice than Yng-F mice but no differences in other age groups. ERβ expression levels in LSK cells were comparable between male and female mice in all age groups. Interestingly, we also found significantly higher AR gene expression in the LSK cells from middle-aged and old female mice than age-matched male mice. Finally, PR gene expression was significantly higher in both Yng-F and Mid-F than the age-matched male LSK cells, and was comparable between LSK cells from Old-F and Old-M mice.

### Distinct sex hormone concentrations in peripheral blood of male and female mice during aging

**Figure 7:**
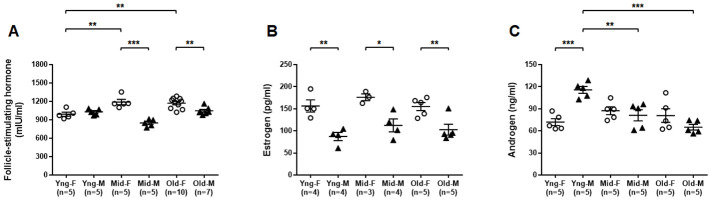
**Comparisons of select sex hormone concentrations in serum between male and female of young, middle-aged and old mice.** Peripheral blood was collected from indicated mice and serum was prepared for ELISA assay. Concentrations of (**A**) follicle-stimulating hormone, (**B**) estrogen and (**C**) androgen in serum between male and female of young, middle-aged and old mice. * P < 0.05, ** P < 0.01, *** P < 0.001.

We used ELISA assays to determine the concentrations of select sex hormones in peripheral blood from both male and female mice of all age groups. Serum FSH concentrations were significantly higher in Mid-F and Old-F mice than Yng-F mice. Serum FSH concentrations were also significantly higher in Mid-F and Old-F mice than age-matched male mice (Figure 7A). Serum estrogen levels were significantly higher in female than age-matched male mice of all age groups (Figure 7B). Serum androgen levels were significantly higher in Yng-M mice than those in Yng-F mice. Serum androgen levels were also significantly higher in Yng-M mice than those in Mid-M or Old-M mice (Figure 7C). Androgen levels were comparable in middle-aged and old mice of both sexes.

### FSH and testosterone promote myeloid differentiation of LSK cells *in vitro*

**Figure 8:**
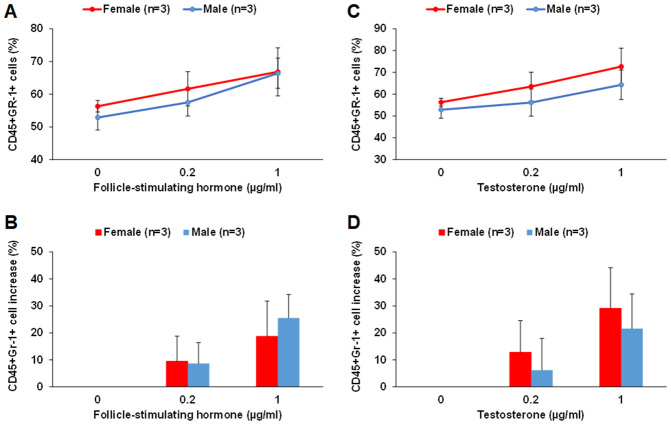
**FSH and testosterone promote myeloid differentiation of middle-aged male and female LSK cells *in vitro*.** (**A** and **B**) FSH at 0.2 and 1 μg/ml concentrations increased the number of CD45+Gr-1+ myeloid cells differentiated from LSK cells of middle-aged male and female mice. The percentage of CD45+Gr-1+ cells among total live cells is shown in (**A**) and the percentage of increase in CD45+Gr-1+ cells with FSH treatment is shown in (**B**). (**C** and **D**) Testosterone at 0.2 and 1 μg/ml concentrations increased the number of CD45+Gr-1+ myeloid cells differentiated from LSK cells of middle-aged male and female mice. The percentage of CD45+Gr-1+ cells among total live cells is shown in (**C**) and the percentage of increase in CD45+Gr-1+ cells with testosterone treatment is shown in (**D**).

By culturing middle-aged male and female LSK cells in the MethoCult GF M3434 methylcellulose medium in the presence of FSH or testosterone, we found that FSH and testosterone could each promote myeloid differentiation of LSK cells *in vitro*. FSH at 0.2 and 1 μg/ml concentrations increased the number of CD45+Gr-1+ myeloid cells by about 8-10% and 20-25%, respectively, for both male and female LSK cells (Figure 8A and 8B). Testosterone at 0.2 and 1 μg/ml concentrations increased the number of CD45+Gr-1+ myeloid cells by about 15% and 30% in female, respectively; about 5% and 20% in male LSK cells (Figure 8C and 8D). There were no differences in CD45+B220+ cells in any of the conditions tested. It is worth noting that despite of our optimization much of the proliferation and differentiation of the LSK cells were already driven by the existing growth factors in the MethoCult GF M3434 methylcellulose medium.

## DISCUSSION

There are substantial sex-biased differences in infection and autoimmunity suggesting that the immune system differs between males and females [30]. Sexual dimorphisms in innate and adaptive immune responses were also shown to contribute to differences in cancer outcome and responses to therapies in men versus women [31]. One hallmark of immune system aging is the functional decline of the immune cells including the HSPCs. Aged HSPCs exhibit increased ground state NF-kB activity which enhances their responsiveness to undergo differentiation and loss of self-renewal in response to inflammation [32]. Physiological aging of the HSPC pool is regulated by mTOR activity and dysregulation of the mTOR pathway in aged mice resulting in their depletion [33] and altered crosstalk between BM-derived endothelial cell niche and HSPCs [34]. Furthermore, accumulation of DNA damage in the aged hematopoietic stem cell compartment has been well established [35]. In addition, epigenetic alterations may also play an important role in hematopoietic stem cell aging [36] and in gender specific HSPCs regulation [37]. Whether intrinsic or extrinsic mechanisms of HSPC aging differ between males and females, and whether these differences may have consequences for disease and lifespan have not been well explored. Our present study shows that there is a significant expansion of BM-derived HSPCs in the middle age of female but in the old age of male mice, whereas previous studies reported a general increase of the HSPC population with aging [35, 38]. Additional studies should reveal if there is also a difference in myelopoietic bias with aging between male and female mice. Our study further reveals that a decline of HSPCs in male mice, as indicated by the expression levels of select hematopoietic genes, occurs much earlier in the aging process than that in female mice. As such, the sexual dimorphism observed on the hematopoietic stem and progenitor cell level may well contribute to the immune system differences between males and females during adult life and aging.

In the current study, we have identified 13 differentially expressed hematopoietic genes in HSPCs, indicated by an asterisk in Tables 1 and 4, which significantly reduced expression in middle-aged male but not in the middle-aged female mice. Both *Fzd1* and its ligand *Wnt3a* are important members of the Wnt/Frizzled signaling pathway, decreased expression of which have been implicated in aberrant inflammatory processes [39] and leukemia development [40]. As the founding member of a family of transcription factors, *Gata1* plays important roles in the development of erythroid and megakaryocyte lineages, and its insufficiencies is involved in primary myelofibrosis and other hematopoietic disorders [41, 42]. Also, *Trim10* is a key factor required for terminal erythroid cell differentiation and survival [43]. *Notch4* and *Dll1* are members of the Notch signaling pathway which, together with Wnt signaling, has been indicated as a possible regulator of adult HSC self-renewal [44]. In addition, a number of other genes, listed in Table 2, were also found to have reduced significantly in old male mice. For example, *Socs5* is highly expressed in long term-HSCs which are known to accumulate with age in males. In the present study however, it is downregulated, which would imply a reduction in long term-HSC frequencies. A recent report suggests that epigenetic silencing of *Socs5* potentiates JAK-STAT signaling and progression of T-cell acute lymphoblastic leukemia [45]. Indeed, male sex is associated with increased risk and poorer survival for age-related leukemia. An earlier study showed that there is a male preponderance among hematological malignancies with a large male excess specific to 60-80 year age group [46]. More recent studies indicated that men have a higher incidence and mortality in chronic lymphocytic leukemia, chronic myeloid leukemia, acute myeloid leukemia and acute lymphocytic leukemia [14, 47, 48]. Overall, male/female incidence ratio in leukemia is 1.66 and in pre-leukemic myelodysplastic syndromes is 1.9 based on a survey of the 2012-2016 Surveillance, Epidemiology, and End Results Program (SEER) data from the National Cancer Institute [49]. However, the cellular and molecular mechanisms underlying sex differences in the incidence, prognosis, and treatment responses of leukemia remain unclear. Hence, there is a critical need to identify cellular and molecular signatures of sex differences in age-related leukemia for the development of sex-specific targeted therapies.

In the sex-mismatched heterochronic BM transplantations, both young male and female LSK cells have acquired an overall higher hematopoietic gene expression level in middle-aged female recipients than in middle-aged male recipients during aging. These results indicate that the middle-aged female BM microenvironment plays a pivotal role in sustaining hematopoietic gene expression during aging. HSPCs are exposed in the BM to a number of cytokines, chemokines and growth factors. It is also known that they respond to certain sex hormones *in vitro*, such as estrogens, androgens and prolactin [26, 50, 51]. Sex hormones exert their biological effects through SexHRs expressed by different types of immune cells. For example, estrogen receptors are present in macrophages, neutrophils, B cells, T cells, natural killer cells, mast cells, and dendritic cells [23, 24, 52]. Androgen receptors are expressed in macrophages, mast cells, and T cells and immature B cells [24, 53]. Indeed, Mierzejewska et al. reported that sex hormones can affect clonogenic growth and differentiation potential of HSPCs [22], and Abdelbaset-Ismail et al. reported that human HSPCs express several functional SexHRs [25]. Hence, sex hormones can influence innate and adoptive immune responses by shaping the development and function of multiple immune cell populations; therefore, they are associated with sex disparities in the immune response leading to susceptibility of infectious diseases, development of autoimmune diseases and cancer. In the present study, we demonstrated that serum estrogen levels were consistently higher in female than age-matched male mice in all age groups. Although it remains possible that the primary female sex hormone estrogen plays a role, it’s unlikely that estrogen is the pivoting factor driving the sexual dimorphism observed here, given that the LSK hematopoietic gene expression profiles of Yng-F vs Yng-M and Old-F vs Old-M were mostly comparable despite the significantly higher serum concentrations of estrogen in female than male mice. Androgen is also a likely factor involved, given that higher androgen receptor expression was found in the HSPCs of both middle-aged and old female mice than age-matched male mice, and comparable androgen serum concentrations were found in mice of these two age groups. Importantly, we found that middle-aged and old female mice have a higher concentration of the pituitary sex hormone FSH in the serum as well as a higher gene expression level of the FSH receptor on the HSPCs than age-matched male mice. This concomitant higher hormone concentration and higher receptor expression of FSH, but not of any other sex hormones, in the middle-aged and old female mice than age-matched male mice suggests that FSH may contribute to the sexual dimorphism in aging hematopoiesis. Indeed, by growing middle-aged male and female LSK cells in the MethoCult GF M3434 methylcellulose medium in the presence of FSH or testosterone, we found that FSH and testosterone could each promote myeloid differentiation of LSK cells *in vitro*. These results are in agreement with earlier studies showing functional expression of several SexHRs on murine HSPCs [22]. Further studies with HSPCs lacking FSH and/or androgen receptors in sex-mismatched heterochronic transplantations are needed to define their roles in the sexual dimorphism during aging hematopoiesis.

In conclusion, our study reveals that the HSPCs in the BM niches are possibly regulated in a sex-specific manner and influenced differently by sex hormones during the aging process. Our study also raises the question as to why a female specific mechanism to delay aging in HSPCs would have evolved, which warrants future investigation. A better understanding of the sexual dimorphism in aging hematopoiesis may allow the development of tailored strategies to maintain optimal immune function for a successful aging in both sexes. Indeed, a recent study by Xu et al. reported that women with premature menopause experience increased odds of developing individual chronic conditions and multimorbidity [54]. Thus, future studies are needed to specifically address sex-effects on the pathogenesis of chronic and autoimmune diseases in the aged populations so that potential differences can be identified and properly addressed therapeutically.

## MATERIALS AND METHODS

### Protocol approval

All experiments with mice have been approved by the Institutional Animal Care and Use Committee (IACUC) of Rhode Island Hospital.

### Mice

Based on the life span and aging criteria of mice set forth by Flurkey et al. [28], we used in this study young adult mice that were 11-13 weeks old, middle-aged mice that were 60-70 weeks old and old mice that are 85-90 weeks old. Both male and female C57BL/6J mice (JAX # 000664) of young and middle-aged groups were purchased from the Jackson laboratory, Bar Harbor, ME. To generate old mice, middle-aged mice were maintained up to 90 weeks at pathogen-free animal facility of Rhode Island Hospital. Both male and female of the C57BL/6 congenic CD45.1 young mice (JAX # 002014, B6.SJL-Ptprca Pepcb/BoyJ) were also purchased from the Jackson laboratory.

### Isolation of lineage-Sca-1+c-kit+ (LSK) hematopoietic stem and progenitor cells (HSPCs)

Total bone marrow (BM) cells were obtained from the femurs and tibias of mice by flushing with PBS containing 2% fetal bovine serum (FBS). We then isolated the HSPC-enriched LSK cell fraction by using a BD Influx flow cytometry cell sorter (BD Biosciences, San Diego, CA) after staining the mouse BM cells with the following antibodies (all purchased from BioLegend, San Diego, CA): Brilliant Violet 421 anti-mouse lineage cocktail, Alexa Fluor 488 anti-mouse Ly-6A/E (Sca-1) antibody and APC anti-mouse CD117 (c-kit) antibody, as described previously [55]. 4’,6-Diamidino-2-Phenylindole (DAPI) was used to exclude DAPI+ dead cells during flow cytometry analysis. Flow cytometry data were analyzed using FlowJo software version 10 (FlowJo, LLC, Ashland, OR). On average 5 x 10^4^ LSK cells were sorted from each mouse and studied as one sample. Samples were not pooled for experiments.

### Hematopoietic gene expression array analysis

Targeted gene array experiments were performed to determine hematopoietic gene expression levels in mouse LSK cells using the RT^2^ profiler PCR array kit for mouse hematopoiesis (Cat. # PAMM-054Z, Qiagen, Waltham, MA). The list of 84 hematopoietic genes in the RT^2^ profiler PCR array can be found at https://geneglobe.qiagen.com/product-groups/rt2-profiler-pcr-arrays, under “Hematopoiesis”. Briefly, total RNA was prepared from the freshly isolated LSK cells immediately after sorting. Each cDNA was synthesized and mixed with the RT^2^ SYBR Green qPCR Mastermix (Qiagen), and then divided in equal volumes to each well of 96 well format array plate. Real time PCR was performed using the CFX 96 Real Time System (Bio-Rad, Hercules, CA). The RT^2^ Profiler PCR Arrays and Assays Data Analysis Program (Qiagen) was used for raw data deposit, organization, analysis, graph drawing and data interpretation. Gene expression of five reference genes, including *β-actin*, *β2M*, *GAPDH*, *β-Glucuronidase*, and *Hsp90ab1*, were used to normalize the expression of hematopoietic genes. Significantly increased or decreased expression level (i.e. change > 2 fold and P < 0.05) between samples was determined. To confirm select results from the hematopoietic gene expression array analysis, we performed real time PCR to determine individual gene expression levels. The following SYBR Green-based QuantiTect Primer Assays were purchased from Qiagen: Mm_*Fzd1* (QT00290542), Mm_*Gata1* (QT00169435), Mm_*Notch4* (QT00135653), Mm_*Il10* (QT00106169), Mm_*Il1a* (QT00113505), Mm_*Trim10* (QT00168385), Mm_*Il12b* (QT00153643), Mm_*Il2* (QT00112315), Mm_*Il11* (QT00122122), Mm_*Mal* (QT00125468), Mm_*Il25* (QT00134645), Mm_*Dll1* (QT00113239), Mm_*Wnt3a* (QT00250439). Reference gene *GAPDH* was used to normalize expression of the hematopoietic genes.

### Sex-mismatched heterochronic BM transplantation

Total BM cells from male and female CD45.1 young donor mice were prepared as described above. BM cell transplantations were performed following twice 5 Gy 3h apart lethal γ-irradiation of middle-aged female or male C57BL/6J recipient mice, where 3 x 10^6^ BM cells were transplanted into each recipient mouse via tail vein injection. To determine donor chimerism in the recipients, peripheral blood samples were collected by retro-orbital bleeding at 16 w post transplantation, and white blood cells were stained with an Alexa Fluor 488 conjugated antibody against CD45.1 (BioLegend). The percentage of CD45.1+ cells in the peripheral blood was analyzed on a BD LSRII flow cytometer and with the FlowJo software as in our earlier studies [55, 56].

### Real time PCR determination of sex hormone receptor (SexHR) gene expression

To determine gene expression of SexHRs, total mRNA was isolated from the LSK cells through Trizol extraction (Thermo Fisher, Waltham, MA). First strand cDNA was synthesized from 1~4 μg total mRNA by using the iScript Advanced cDNA synthesis Kit for RT-qPCR (Bio-Rad). Different SexHR and internal control (*GAPDH*) mRNA levels were detected by using real time PCR with the iTaq Universal SYBR Green Supermix (Bio-Rad) on the CFX 96 Real Time System (Bio-Rad). PCR primers for the SexHRs were synthesized by Integrated DNA Technologies (Coralville, IA) based on the primer sequences published elsewhere [22]. *GAPDH* expression was assessed as an internal reference for quantification. Forward and reverse primers for *GAPDH* were as follows: 5’ - AAA AGC AAC TCC CAC TCT TC - 3’ and 5’ – CCT GTT GCT GTA GCC GTA TT - 3’. Relative gene expression level of SexHRs was expressed by the fold increase over internal control samples. Every real time PCR was performed in triplicates.

### Enzyme-linked immunosorbent assay (ELISA)

Peripheral blood samples of 100 μl were collected from the retro-orbital sinus of young, middle-aged, and old male and female mice. Blood was allowed to clot for 20 min at room temperature. The supernatants were collected after centrifugation, and 10 μl serum were used for each sample. FSH, estrogen and androgen levels in each serum sample were determined by using ELISA kits (Mybiosource, San Diego, CA) according to manufacturer’s instructions. The plates were read using a Spectra Max 190 microplate reader (Molecular Devices, Sunnyvale, CA) at 450 nm wavelength. Sex hormone concentration in serum was determined as mIU/ml for FSH, pg/ml for estrogen and ng/ml for androgen.

### *in vitro* clonogenic assay of LSK cells in the presence of FSH or testosterone

BM-derived LSKs were obtained from middle-aged female or male mice per flow cytometry cell sorting as described above. In order to assay the potential effects of FSH and testosterone, the original growth factor concentrations in the MethoCult GF M3434 methylcellulose medium (STEMCELL Technologies) were halved by mixing with the MethoCult M3234 medium at 1:1 ratio. LSK cells (5000 cells/well) were then resuspended in 1.5 ml of the growth factor-reduced culture medium containing mouse recombinant FSH (R and D Systems, 0.2 or 1 μg/ml) or testosterone (Sigma-Aldrich, 0.2 or 1 μg/ml). The length of the *in vitro* clonogenic assay with FSH or testosterone was optimized in separated preliminary experiments. Total cells were harvested after 9 days of culture, and colony forming and differentiation were evaluated under a microscope and then analyzed using flow cytometry after staining with anti-mouse antibodies against CD45 (for all hematopoietic cells), Gr-1 (for myeloid cells), and B220 (for B cells). Each experiment was performed multiple times in duplicates.

### Statistical analysis

Data are shown as the mean values ± standard error of the mean (SEM). The significance of difference was calculated with unpaired two-tailed Student’s t-test or one-way ANOVA when comparing more than 2 groups by using Microsoft Office Excel (Redmond, WA) or the GraphPad Prism (GraphPad Software, Inc., La Jolla, CA). P values less than 0.05 were considered to be statistically significant.
